# Exploratory EEG-TMS Study Reveals Altered Behavioral Function in Individuals Following Anterior Cruciate Ligament Reconstruction

**DOI:** 10.3390/brainsci16020156

**Published:** 2026-01-29

**Authors:** Haley R. Huntington, Christine E. Phelps, Tim Lehmann, Daniel Büchel, Anika Khurana, Louis Y. Wang, Anisha A. Patel, Caitlyn E. Olshausen, Lana J. Kayali, Tina Boluordi, Maelani Nguyen, Yong Woo An

**Affiliations:** 1 College of Science & Health, Charles R. Drew University, Los Angeles, CA 90059, USA; haleyhuntington@cdrewu.edu; 2 Department of Health & Human Sciences, Loyola Marymount University, Los Angeles, CA 90045, USA; christinephelps03@gmail.com (C.E.P.); anikakhurana24@gmail.com (A.K.); wanglouis0211@gmail.com (L.Y.W.); apatel40@lion.lmu.edu (A.A.P.); caitie.olshausen@gmail.com (C.E.O.); lkayali@lion.lmu.edu (L.J.K.); tboluord@lion.lmu.edu (T.B.); mnguye78@lion.lmu.edu (M.N.); 3 Exercise Science & Neuroscience, Department Exercise & Health, Paderborn University, 33098 Paderborn, Germany; tim.lehmann@uni-paderborn.de (T.L.); daniel.buechel@uni-paderborn.de (D.B.)

**Keywords:** ACL reconstructive surgery, ACL injury, neuroadaptation, corticospinal excitability, TMS

## Abstract

Background: Following anterior cruciate ligament reconstruction (ACLR), ACLR patients often experience quadriceps dysfunction, potentially linked to increased corticospinal excitability. However, the role of motor cortex neuroadaptations in persistent quadriceps strength deficits remains unclear. Purpose: The purpose of this study is to investigate neural behavior during a force reproduction task using transcranial magnetic stimulation (TMS) in ACLR participants compared to healthy controls (CONT). Methods: Electrocortical activation of 16 ACLR (10F and 6M, 20.0 ± 1.2 years, 171.9 ± 7.2 cm, 75.8 ± 17.1 kg) and 16 CONT (10F and 6M, 20.6 ± 1.4 yrs, 168.0 ± 9.9 cm, 66.3 ± 11.0 kg) was measured using a 64-channel EEG system during an isometric force reproduction task. Sixty TMS pulses (≥120% active motor threshold) were delivered to the primary motor cortex while participants maintained 10% of quadriceps maximal voluntary isometric contraction (QMVIC10%). Motor-evoked torque (METnorm, %), normalized to 100% TMS intensity, was measured to assess neuroadaptation in the corticospinal tract. EEG data was processed to compute N100 (80–200 ms) and P200 (160–300 ms) TMS-evoked event-related potentials (TEPs, µV) at three regions of interest (ROI): the motor (ROI1), parietal (ROI2), and frontal (ROI3) cortices. MET and TEP comparisons were conducted using independent and unpaired two-sample permutation-based *t*-tests, respectively. Results: The ACLR group exhibited a significantly greater MET than CONT. Although exploratory, differences were found in P200 TEP at ROI1 with lower power in ACLR than CONT. Conclusions: Lower TEP amplitude at ROI1 implies neural inhibition in the motor cortex, while heightened MET in ACLR suggests greater corticospinal excitability. Neural adaptations in the corticospinal tract in ACLR patients may contribute to excessive quadriceps activation in response to unanticipated stimuli, potentially increasing the risk of re-injury.

## 1. Introduction

Every year, thousands of patients worldwide undergo reconstructive surgery of the anterior cruciate ligament (ACL) [1], a key structure for maintaining knee mechanical stability [2]. Despite advancements in surgical procedures and rehabilitation protocols, many patients continue to experience persistent knee dysfunction following ACL reconstruction (ACLR). These deficits may present as chronic pain, reduced range of motion, quadriceps weakness, impaired voluntary contraction, increased risk of reinjury, and early-onset osteoarthritis [2,3]. Among these impairments, persistent quadriceps weakness and difficulty controlling force output are relevant to functional performance following ACLR.

Quadriceps weakness after ACLR is often attributed to arthrogenic muscle inhibition (AMI), a reflexive neural mechanism in which the nervous system temporarily suppresses muscle activation to protect the injured joint [4]. Although AMI is adaptive in the acute phase, persistent AMI may lead to long-term weakness and functional limitations. Emerging evidence suggests that AMI involves not only peripheral inhibition but also central alterations, including changes in resting motor thresholds and atypical cortical activity [5]. Thus, sustained AMI may compromise quadriceps activation and drive neuroplastic changes within the corticospinal pathway [4,6].

Several TMS studies have indicated that persistent quadriceps deficits following ACLR can be linked to neural adaptation within the corticospinal pathway [7,8]. ACLR patients with quadriceps weakness often demonstrate significantly higher active motor thresholds (AMT) in the injured limb [1,7,9], suggesting a persistent reduction in corticospinal excitability. This decreased excitability may result from increased inhibitory interneuron activity at both the spinal cord and motor cortical levels, necessitating a greater stimulus to activate the quadriceps [10]. However, increased motor-evoked potentials (MEPs) have also been observed in ACLR patients, indicating enhanced corticospinal output during voluntary quadriceps activation. This pattern of reduced excitability at rest but greater MEPs during quadriceps contraction may reflect compensatory neuroplastic adaptations within the corticospinal tract as well as in additional cortical regions, such as the primary motor (M1), premotor, and somatosensory cortices [11,12]. These additional neural resources may be recruited to overcome persistent quadriceps inhibition, thereby facilitating motor output despite ongoing deficits at the spinal–motor pathway [13].

Neuroimaging studies have also suggested that neural adaptation in brain regions, including the somatosensory and frontal cortices, may contribute to both abnormal motor patterns and an increased risk of ACL retear [14,15]. However, these adaptations may also serve a protective compensatory mechanism to process altered sensory input from the ACL-reconstructed knee and help maintain normal knee function post-recovery [13]. Consequently, the primary motor and secondary somatosensory cortices have shown greater activation in ACLR patients during knee flexion and extension tasks, likely reflecting increased cortical drive requirements during quadriceps contraction [13]. Given that the primary motor cortex is essential for planning and executing voluntary movements [16], ACLR patients with altered corticospinal excitability may need enhanced neural drive from this region to achieve proper quadriceps contraction [17].

When combined with TMS, alterations in cortical excitability can be effectively investigated, as induced involuntary muscle contractions produce measurable motor-evoked torque (MET) in the corresponding motor areas of the cortex [18]. Additionally, TMS pulses to the motor cortex can instantly elicit neural activation in the adjacent frontal and parietal cortices, potentially altering motor function [19]. Mobile electroencephalography (EEG), with its millisecond-level temporal precision, allows for the detection of immediate neural changes following stimuli such as transcranial magnetic stimulation (TMS) [20]. Therefore, the combination of TMS and EEG techniques can provide insight into neuroadaptation related to quadriceps deficits following ACLR.

Event-related potentials (ERPs), measured from EEG, are time-locked electrical potentials that reflect the brain’s responses to sensory, cognitive, or motor events [20]. ERPs are characterized by their distinctive waveform components corresponding to different stages of neural processing, thereby providing insight into our sensory and cognitive cortical functions [20,21]. Furthermore, research suggests that such potentials can explain the brain’s adaptive responses to altered motor demands following injury [21].

TMS-evoked potentials (TEPs) are ERPs induced by a TMS pulse and can be used to assess cortical responsiveness and intercortical connectivity on a millisecond scale [22,23]. TEPs arise from direct cortical activation following a TMS pulse and reflect cortico-cortical pathways and changes in cortical excitability [24,25,26]. Following stimulation of the primary motor cortex (M1), early TEP components occurring within the first 20 to 60 ms post-stimulation, such as the P25, P30, N45, and P60, are considered primary markers of local M1 and adjacent somatosensory activation and are present in central areas of the brain [23,27,28]. However, TEPs elicited by motor cortex stimulation are often coupled with corticospinal tract excitation, making it difficult to determine whether the brain or the cortical tract is more responsible for the muscle contraction [22]. In particular, later TEP components such as the N100 and P200 exhibit more widespread scalp distributions across the frontal and parietal cortex and are influenced by auditory and somatosensory co-stimulation and processing associated with the TMS pulse [23,28]. Accordingly, they are considered mixed sensorimotor potentials rather than purely local indices of M1 excitability [22]. As a result, different TEP components are evaluated within the context of motor control and sensorimotor integration, with later components providing insight into distributed and systems-level processing during task-related perturbations. Interestingly, the pathways involved in TEPs represent brain areas often associated with neuroplastic changes following ACL injury [29]. Therefore, TEPs appear to be a promising approach to investigate neuroplasticity following ACL injury.

The N100 TEP component is a negative deflection around 100 ms post-stimulation, generated by a network of neurons responding to unexpected stimuli and varying in latency and amplitude depending on the brain region and sensory modality. The N100 component of the motor cortex TEP is widely interpreted as a marker of GABA-B-mediated intracortical inhibition, a slow, long-lasting form of neuro-inhibitory control that contributes to the regulation of cortical excitability and network-level dynamics [30,31]. However, it is a combination of inhibitory function and non-specific sensory inputs, and can be strongly influenced by auditory and somatosensory inputs evoked by the TMS pulse. P180/P200 is thought to reflect late, interconnective functions of reafferent sensory inputs and network-level cortical activity [23,24] This component is also associated with perceptual processing and is associated with attentional engagement [32]. Accordingly, within the context of TMS-pulse disruptions within an isometric force reproduction task, analysis of late TEP components informs a systems-level integration of sensorimotor inputs and motor outputs, in contrast to early components that are more specific to M1 and somatosensory system activation [23]. They can therefore allow the identification of differences in neural activation patterns between ACLR patients and healthy controls (CONT), highlighting complex neural interactions and potentially providing invaluable insights for effective rehabilitation strategies [33]. Although persistent quadriceps strength deficits are often attributed to neuromuscular changes, the specific influence of neural changes on motor control remains unclear. While altered corticospinal excitability has been documented in ACLR patients, the relationship between neuroadaptations in the corticospinal tract and primary motor cortex has not been extensively investigated [34]. Therefore, the purpose of this study was to examine corticospinal tract and brain activation patterns in ACLR participants during a force reproduction task. Comparing ACLR and CONT participants’ neural patterns during isometric tasks can enhance understanding of how ACL injury affects the corticospinal pathway and primary motor cortex. Additionally, examining correlations between components in each group can inform us about the presence and/or strength of a relationship, providing an important understanding of interactions between the corticospinal tract and motor cortex. We hypothesized that ACLR participants would exhibit higher motor-evoked torque (MET) and lower TEP peak power compared to CONT, reflecting increased cortical excitability in ACLR, as indicated in prior studies. Heightened quadriceps activation with TMS in ACLR participants may only reflect neuroadaptation at the corticospinal tract, rather than the motor cortex. Therefore, it is necessary to assess both MET and TEP components in frontal, central, and parietal brain areas following reconstructive surgery to better understand the extent of the neuroadaptations in the corticospinal tract and motor cortex. By analyzing brain activation patterns in neuromuscular control, especially during force reproduction tasks, this study aims to improve our understanding of neural behavior following ACL injury and support the development of advanced rehabilitation strategies post-ACLR.

## 2. Materials and Methods

### 2.1. Participants

Sixteen participants with ACLR (10 females, 6 males, 20.0 ± 1.2 years, 171.9 ± 7.2 cm, 75.8 ± 17.1 kg) and sixteen healthy controls (10 females, 6 males, 20.6 ± 1.4 years, 168.0 ± 9.9 cm, 66.3 ± 11.0 kg) volunteered to participate in this study (Table 1). The sample size was calculated using an a priori power analysis based on a published TMS-EEG study for a force reproduction task [12]. As a result, the minimum sample size to detect a moderate effect size at a probability of 0.05 with 80% power required nine participants for each group. Participants between groups were matched in gender, age, and participating legs for analysis in this case-control study. Individuals in the control group (CONT) had no history of neuromuscular impairments or undergone reconstruction surgery, while the ACLR group must have had reconstruction surgery at least 6 months prior to the study and have been cleared by a physician to exercise again (21.71 ± 22.36 months). Participants with prior neurological or knee injuries were screened to ensure participatory qualification. Researchers informed all participants of the study protocols, the voluntary nature of this research, and their right to cease participation in this study at any time. Participants read and signed an informed consent form approved by the Institutional Review Board at Loyola Marymount University for participation in this study. This study is exploratory in nature, which is common for initial investigations into a novel topic using complex methodology such as TMS-EEG.

### 2.2. Surveys

This study utilized a compilation of surveys that gathered details about the participants’ demographics (e.g., sex, age, height, weight), medical history (e.g., mechanism of injury during ACL sprain, graft type used for reconstruction surgery, previous musculoskeletal injuries, and neurological medical conditions, etc.), and scores from standardized tools. Three self-reported questionnaires were also employed to determine overall knee function at the time of data collection. The International Knee Documentation Committee (IKDC) Subjective Knee Evaluation was used to measure symptoms, functions, and sport activity levels for patients with preexisting knee conditions [35], The Lysholm Knee score measured knee instability after treatment [35], and the Tegner Activity Level Score evaluated the participants’ activity levels before and after injury [36].

### 2.3. Pre-Testing Setup

Electrocortical activation was recorded using a 64-channel waveguard™ EEG cap at 1 KHz sampling rate (eegoTM Sports, ANT Neuro, Hengelo, The Netherlands). The cap was centered on the head according to the international 10–20 placement system [37], including CPz as the reference and FCz as the ground electrode. Electroconductive gel was used to ensure a signal impedance of less than 20 kΩ. Ear plugs were provided to participants to reduce the volume of TMS-click when TMS stimulation was elicited.

**Figure 1:**
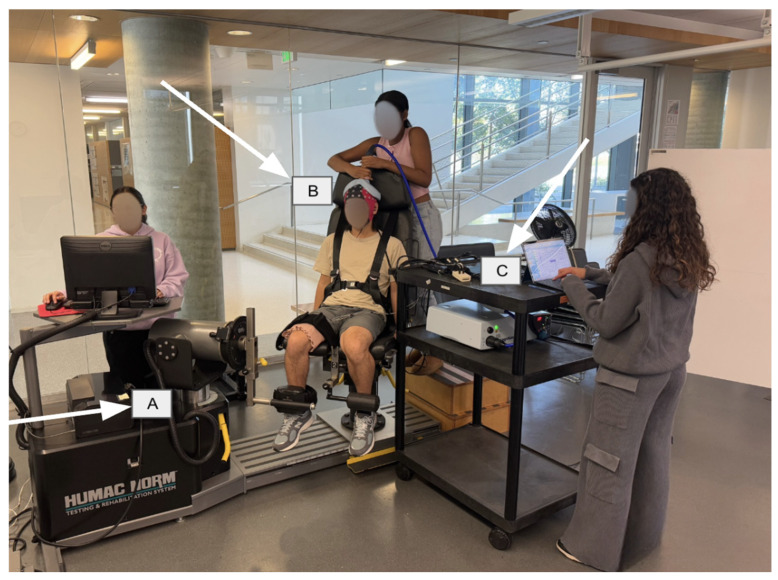
Displays set-up used in the protocol. The participant is positioned on the HUMAC Dynamometer, with the 64-channel electrode cap on the head. (A) HUMAC NORM dynamometer. (B) TMS coil. (C) EEG acquisition.

The participant was then seated on a HUMAC Norm isokinetic dynamometer (Computer Sports Medicine, Inc., Stoughton, MA, USA) with the testing knee at 90° flexion and trunk at 85° flexion. To isolate quadriceps activation, participants were positioned with 85° trunk flexion, secured by a seatbelt, and 90° knee flexion of the tested limb, stabilized with Velcro straps around the quadriceps and ankle. The contralateral leg was positioned behind a lever, ensuring focused activation of the testing quadriceps (Figure 1). Correct positioning on the HUMAC ensures the lateral femoral epicondyle is in line with the fulcrum of the HUMAC Dynamometer arm.

In this study, we employed MET rather than MEP amplitude, as MEPs are highly variable and influenced by several factors, including arousal level, muscle state, and intrinsic fluctuations [38]. In contrast, MET provides a more reliable measure of corticospinal excitability, with an excellent reliability (ICC = 0.90 at >110% AMT) [18]. As the first study combining EEG, TMS, and ACLR populations during a submaximal isometric contraction task, our primary aim was to evaluate the general cortical response of ACLR patients to TMS pulses, for which MET represented the most appropriate and reliable approach.

While participants were seated in the HUMAC Dynamometer, hotspot detection was performed following established TMS protocols [39]. Stimulation began at an initial spot marked on the EEG cap 2 cm posterior and 2 cm lateral to the Cz electrode, with four additional spots 1 cm each to anterior, posterior, lateral, and medial to the initial spot. TMS pulses at 30% intensity were delivered to these five spots, while participants maintained 10% of their quadriceps maximum voluntary isometric contraction (QMVIC). The hotspot was then identified as the location among these five spots that produced the greatest and most consistent knee extension twitch torque in response to TMS pulses delivered via a double-cone TMS coil (Magstim Company, Wales, UK). If significant quadriceps twitch torque was not observed, the hotspot detection procedure was repeated with 5% increasing intensity increments [18]. Active motor threshold (AMT_%MSO_, %) was then determined as the minimal TMS intensity required to produce the largest and most consistent knee twitch torque using the TMS Motor Threshold Assessment Tool (MTAT 2.1) and the Adaptive PEST procedure [40]. This threshold value was then used for the remainder of the experiment.

### 2.4. Testing Protocol

**Figure 2:**
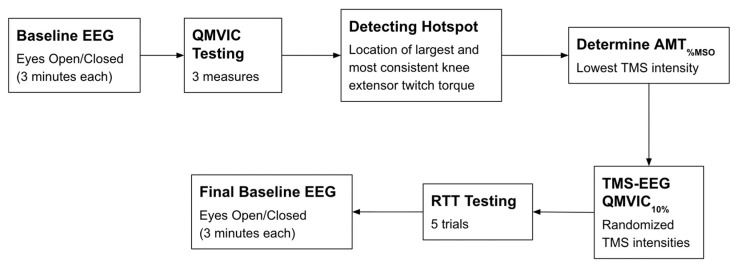
Schematic of the experimental protocol. Abbreviations: EEG: electroencephalography; QMVIC: Quadriceps Maximal Voluntary Isometric Contraction; AMT_%MSO_: Active motor threshold in which the minimum TMS intensity is applied to produce the largest and consistent METs; TMS: transcranial magnetic stimulation; RTT: resting twitch torque.

A schematic of the experimental design is provided in Figure 2. After 5 min of warm-up on a stationary bike, participants performed two practice trials followed by three measurements of QMVIC. For the QMVIC measurements, participants exerted maximum force against the dynamometer arm while they received verbal cues from investigators and visual feedback of the torque curve on the HUMAC2015 software (300904, Computer Sports Medicine, Inc., Stoughton, MA, USA). A one-minute rest interval was implemented between each trial to mitigate muscle fatigue. Ten percent of the average QMVIC was calculated and utilized for the remaining protocol.

### 2.5. TMS Parameters

Both 120% AMT and 140% AMT have been demonstrated to effectively and consistently elicit muscle twitch torque responses in individuals with pathologies [41]. Given that MET amplitudes can vary considerably among individuals, AMT at a higher percentage may be necessary to ensure a consistent elicited response from the TMS [42]. Therefore, 60 TMS pulses at either 120% AMT (30 trials) and 140% AMT (30 trials) were randomly delivered to the hotspot to conduct knee extensor twitch torques. Lastly, five trials of resting twitch torque (RTT) were conducted by applying TMS pulses at 100% intensity directly over the quadriceps, positioned 20 cm proximal to the patella [18].

### 2.6. EEG and Quadriceps Strength Data Processing

To process the EEG data, the TMS-EEG signal analyzer (TESA) [43] plugin for EEGLAB v2023.1 [44] was used in MATLAB (R2023a, v9.14, MathWorks Inc., Natick, MA, USA). First, TMS pulses were detected, and the EEG recordings were divided into segments of 12 s with respect to the TMS onset [−6 s to 6 s]. Then, baseline removal was performed for each epoch based on the time period between −6 s and 0 (TMS onset). To remove large-value magnetic field noise resulting from the TMS pulse, the data between 0 and 20 ms was removed in each epoch. For handling the resulting missing data points in the EEG recordings, linear interpolation was applied using the tesa_interpdata function. This function fits a linear model based on the data points preceding and following each missing value and replaces the missing data correspondingly. This approach assumes a linear relationship between neighboring data points, ensuring that gaps in the data do not affect subsequent analyses. Subsequently, the data was downsampled to 500 Hz, and the fast independent component analysis (fastICA) was performed to decompose the signal into maximally independent components. Based on the fastICA decomposition, the pop_tesa_compselect function was used to remove components containing TMS-induced muscle artifacts, ocular artifacts, muscle activity, and electrical noise, following the approach described by Rogasch et al. [43,45]. Automatic identification of artifactual components was carried out using the pop_tesa_compselect function, which applies a series of quantitative criteria to distinguish different types of artifacts. To detect TMS-evoked muscle activity, the method compared the mean absolute amplitude of each component’s time course within a predefined post-stimulation window to its mean absolute amplitude across the entire epoch. Components were flagged as artifactual if the amplitude within the targeted window exceeded the global mean by at least a factor of eight, reflecting a substantial transient increase in muscle activity following TMS. Eye blink artifacts were identified by evaluating the mean absolute z-score of component spatial weights at prefrontal electrodes (Fp1, Fp2). If both electrodes demonstrated a mean z-score greater than 2.5, the component was marked as a likely blink-related artifact. Lateral eye movement artifacts were detected by examining the z-scores at lateral frontal electrodes (F7, F8), with criteria requiring the z-score to exceed +2 at one electrode and be less than −2 at the contralateral site, capturing the opposing topography characteristic of saccadic movements. Detection of components contaminated by persistent muscle activity was based on the slope of the regression line fitted to the log-transformed power spectrum of the component, following the approach outlined by Fitzgibbon et al. (2016) [46]. Finally, components representing electrode noise were flagged if any single electrode’s spatial weight had an absolute z-score of at least 4, indicating focal, non-neurogenic activity.

After the independent component removal, a fourth-order butterworth filter with a bandpass from 3–50 Hz was applied. Finally, the tesa_peakanalysis function was used to perform analysis of the TEPs in three a priori defined midline regions of interest (ROIs), further referred to as motor (ROI1: C1, Cz, C2), parietal (ROI2: P1, Pz, P2), and frontal (ROI3: FC1, FCz, FC2) ROIs. These clusters were chosen a priori based on standard 10–20 locations over primary motor, parietal, and frontal cortices and on previous TMS-EEG literature using similar midline “vertex” or central ROIs to quantify N100-P200 responses following M1 stimulation [47,48]. This anatomically constrained ROI approach was preferred over data-driven, effect-based channel selection to avoid circularity and to limit multiple comparisons in this pilot study. Midline ROIs in the frontal, central, and parietal cortices, revealed from current evidence of the scalp topographies of grad average responses, typically reveal dynamic topographies in early (temporal), medium (frontal), and late (phases) following stimulation. The adoption of a region of interest (ROI) approach over single-channel analysis is justified by its efficacy in enhancing the signal-to-noise ratio (SNR) and providing a more robust measure of localized neural activity. This method leverages the principle of spatial averaging across a cluster of proximate electrodes. For source space analyses, the number of data points was not sufficient because of the limited number of stimulations performed.

**Figure 3:**
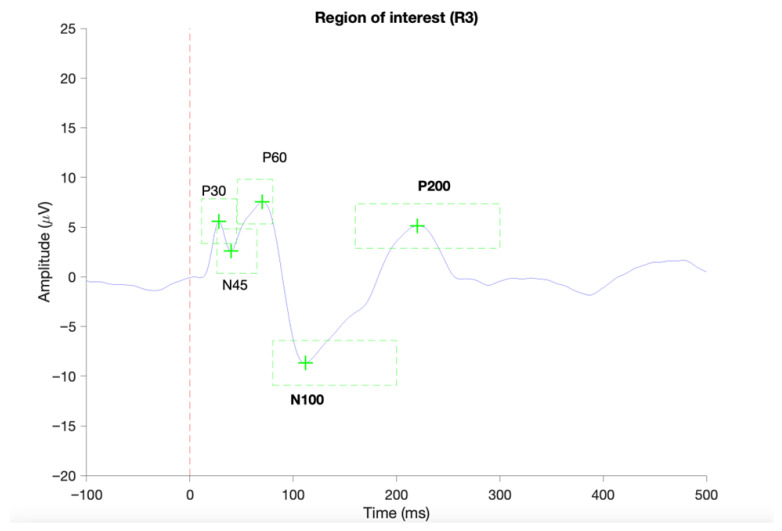
Provides an example of a successful identification of TEP components in ROI 1 (C1, Cz, C2) based on average TEP of a single subject using the TESA toolbox [43]. The red vertical dashed line indicated the onset of the TMS pulse. Green dashed boxes indicate the predefined time periods for the identification of local negative or positive peaks.

**Figure 4:**
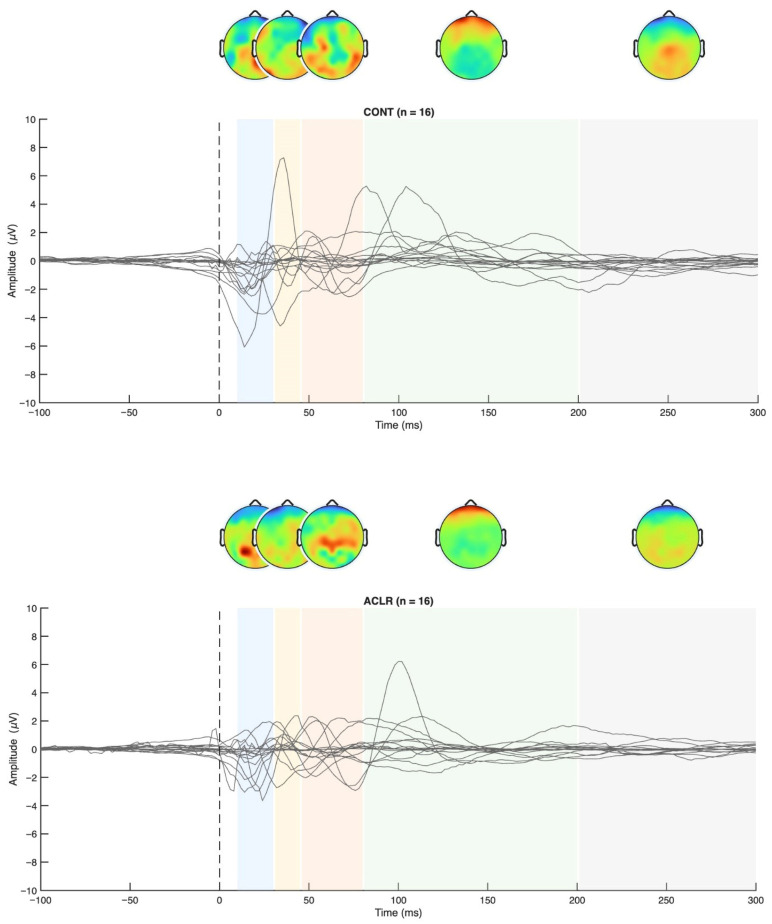
TEP grand averages for both groups and topographical maps.

The TEPs were scanned for predefined components, namely the P30 (11–45 ms), N45 (26–65 ms), P60 (46–80 ms), N100 (80–200 ms), and P200 (160–300 ms). In case of identification of local positive or negative peaks in the predefined time-windows of interest, the mean TEP components amplitudes and latencies were extracted for the individual subjects. When TEP component detection failed, subjects were excluded from the given TEP component statistics. An example of a complete identification of TEP components is provided in Figure 3. Although extensive artifact correction, including ICA-based removal of ocular components and rejection of high-amplitude epochs, was applied, visual inspection of the grand-average butterfly plots (Figure 4) indicated that some residual non-neural activity (i.e., eye blinks, small eye movements, and muscle activity) was still present, particularly in channels showing large deflections. For this reason, butterfly plots and topographies were used only as qualitative visualization tools and results were interpreted with caution.

**Figure 5:**
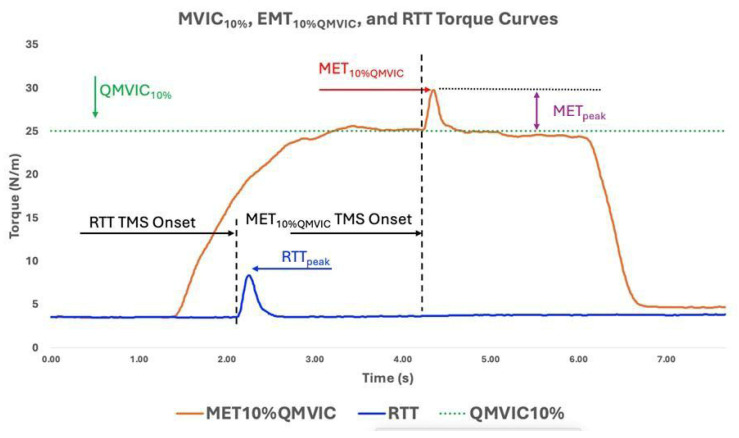
Displays QMVIC_10%_, MET_10%QMVIC_, and RTT torque curves for one ACLR participant. The Utt_arak_hand MET_10%QMVIC_ and MET_peak_ display the peak torque value in response to TMS stimuli to the motor cortex.

As behavioral parameters, averaged QMVIC (Nm), QMVIC normalized to body weight (QMVIC_norm_, Nm/kg), 10% of QMVIC (QMVIC_10%_, Nm), minimum TMS intensity to produce largest and consistent METs (AMT_%MSO_, %), MET peak torque amplitude to TMS stimuli (MET_10%QMVIC_, Nm), absolute MET torque increase from QMVIC_10%_ (MET_peak_, Nm, Equation (1)), MET peak torque latency (MET_lat_, ms), peak resting twitch torque (RTT_peak_, Nm), and MET normalized to RTT_peak_ (MET_norm_, Equation (2)) were calculated and reported for further statistical analysis. An example of MET_10%QMVIC_, RTT_peak_, and QMVIC_10%_ curves is provided in Figure 5.

(1) $${MET}_{peak}\;\left(Nm\right)={MET}_{10\%QMVIC}-{QMVIC}_{10\%}$$

(2) $${MET}_{norm}\;\left(\%\right)\;=\frac{{MET}_{peak}\;\left(Nm\right)}{{RTT}_{peak}\;(Nm)}$$

### 2.7. Statistical Analysis

Normality of the data was evaluated using the Shapiro–Wilk test, and QMVIC, QMVIC_norm_, AMT_%MSO_, MET_10%QMVIC_, MET_peak_, MET_lat_, RTT_peak_, and MET_norm_ variables followed a normal distribution. However, several knee function outcome measures (IKDC, Lysholm, Tegner) violated normal distribution. Therefore, the Mann–Whitney U test was conducted to further observe differences between ACLR and CONT in these knee function outcomes. Levene’s Test for Equality of Variances confirmed no significant differences in height, weight, and age variances between groups. Statistical analysis of TEP components was performed using customized scripts in MATLAB. Due to the limited number of trials per TMS pulse intensity, successful TMS pulses were pooled from both AMT120% and AMT140% for TEP-analysis. Outliers in TEP component amplitudes and latencies were identified as values exceeding three scaled MADs from the median and were removed. An unpaired two-sample permutation-based *t*-test (*n* = 2000 permutations)—based on the t-statistic of the hypothesis that the data in the CONT and ACLR comes from distributions with equal means—was performed (*p* < 0.05). The resulting *p*-values and confidence intervals (CI) were adjusted for multiple comparisons using the max correction method [49]. Statistical analysis of MET was performed using SPSS v24.0 (Statistical Package for Social Sciences; SPSS Inc., Chicago, IL, USA). Independent *t*-tests were performed for quadriceps strength data including QMVIC, QMVIC_norm_, AMT_%MSO_, MET_10%QMVIC_, MET_peak_, MET_lat_, RTT_peak_, and MET_norm_. A statistical significance level of 0.05 was set for all analysis. Effect size, interpreted as small (η^2^ = 0.01, d = 0.2, r = 0.1), medium (η^2^ = 0.06, d = 0.5, r = 0.3), and large (η^2^ = 0.14, d = 0.8, r = 0.5), with a confidence interval of 95% indicating significance, were evaluated to establish the practicality of our findings [50]. Spearman’s correlation was also used to analyze interactions between quadriceps twitch torque values and TEP components. Because MET_norm_ is calculated from MET_peak_ and RTT_peak_, additional correlation and covariance analysis were performed to determine whether RTT_peak_ influenced MET_norm_ values. Pearson correlations were performed between RTT_peak_, MET_peak_, and MET_norm_. An ANCOVA was also conducted with MET_norm_ as the dependent variable, Group as the fixed factor, and RTT_peak_ as a covariate to evaluate whether group differences in MET_norm_ persisted after accounting for variability in RTT_peak_. Estimated marginal means and pairwise comparisons were examined to determine adjusted group differences.

## 3. Results

### 3.1. Quadriceps Strength Analysis

**Figure 6:**
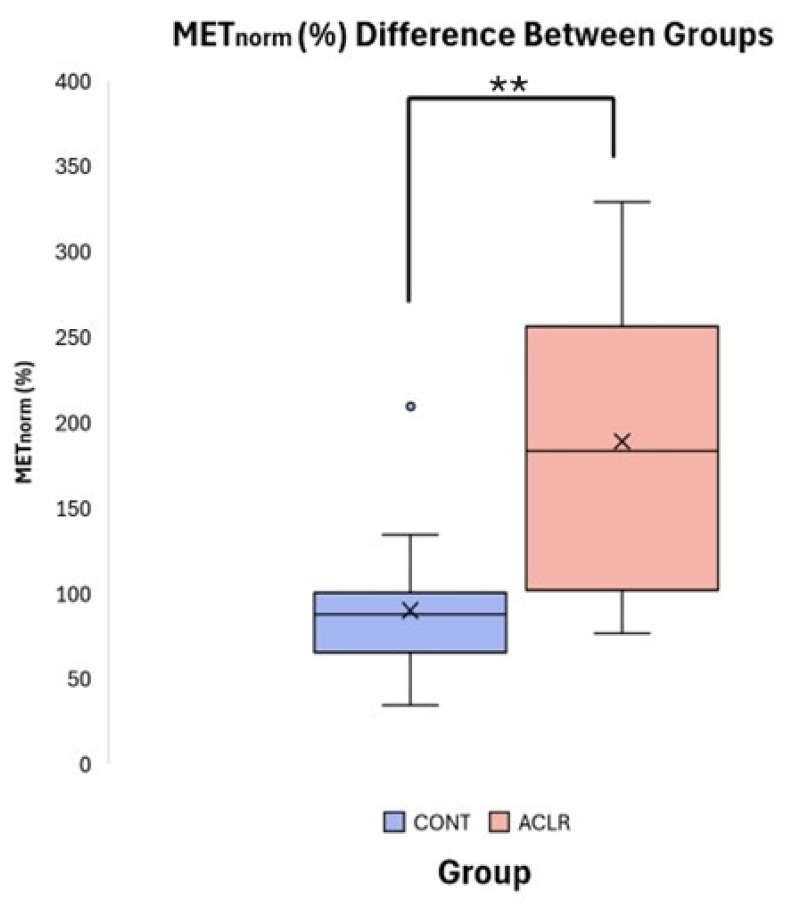
Boxplot comparison of MET_norm_ (%) between CONT and ACLR. (** denotes a significant difference between groups *p* < 0.001).

Independent *t*-tests revealed significant group differences for MET_peak_ (t = 3.24, df = 30, *p* = 0.003, Cohen’s d = 1.14, 95% CI [0.37, 1.86]) and MET_norm_ (t = 4.180, df = 21.104, *p* < 0.001, Cohen’s d = 1.48, 95% CI [−0.66, −2.22]). The ACLR group (MET_peak_: 12.24 ± 3.76 Nm, MET_norm_: 188.97 ± 85.94%) exhibited significantly greater involuntary quadriceps activation compared to the CONT group (MET_peak_: 8.08 ± 3.52 Nm, MET_norm_: 90.08 ± 39.63%) following unanticipated TMS pulse delivery to the primary motor cortex (Table 2, Figure 6). Pearson correlation analyses indicated that RTT_peak_ was not significantly associated with MET_peak_ (r = 0.269, *p* = 0.136), but demonstrated the expected moderate negative association with MET_norm_ (r = −0.533, *p* = 0.002), due to normalization. However, ANCOVA revealed that the group difference in MET_norm_ remained significant after adjusting for RTT_peak_ (f [1, 9] = 15.091, *p* < 0.001, partial η^2^ = 0.342). Adjusted estimated marginal means were 98.0% for the CONT group and 181.0% for the ACLR group, demonstrating that the observed group difference in MET_norm_ was not biased to RTT_peak_.

### 3.2. Knee Function Outcomes and Correlation Data 

**Figure 7:**
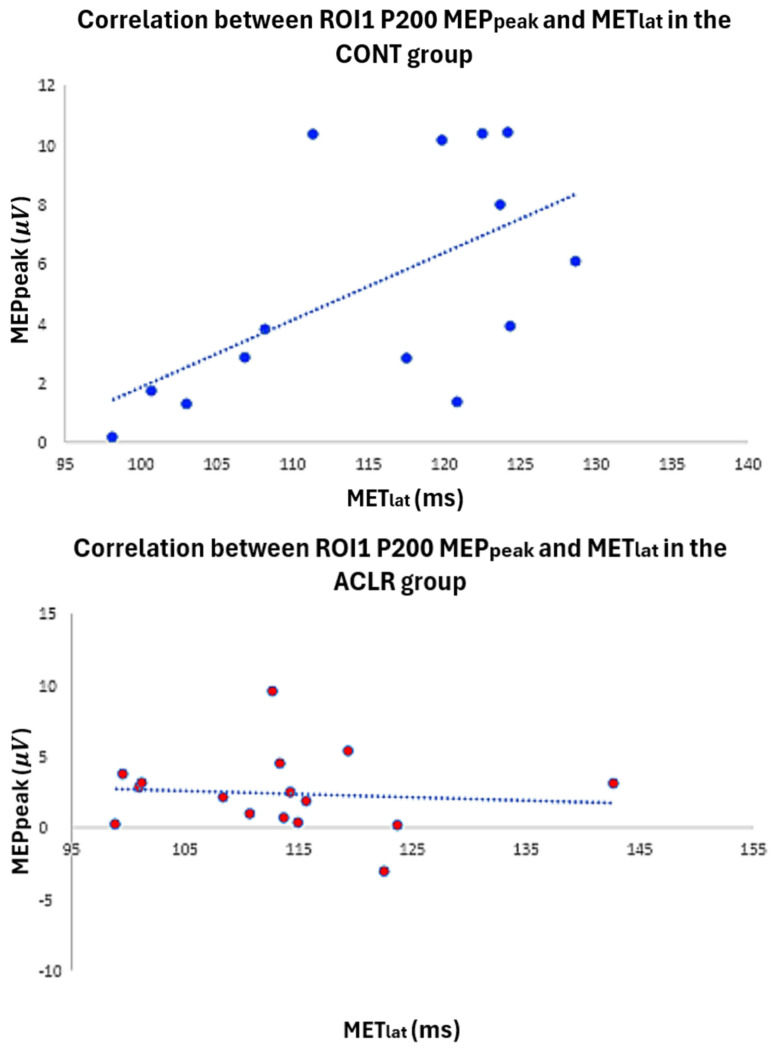
Scatter plot illustrates the relationship between P200 latency and MET latency in both the control and ACLR groups.

According to the Mann–Whitney U test (Table 1), the ACLR group demonstrated significantly lower scores for the IKDC (ACLR: 8.84, CONT: 24.16, *p* < 0.001, U = 5.50, 95% CI [9.20, 17.24]) and Lysholm (ACLR: 9.5, CONT: 23.5, *p* < 0.001, U = 16.00, 95% CI [5.00, 19.00]) than the CONT group. However, there was no significant difference in Tegner scores between groups (ACLR: 15.47, CONT: 17.53, *p* = 0.525, U = 111.50, 95% CI [−1.00, 2.00]). In the CONT group, a significant moderate positive correlation (*ρ* = 0.587, *p* = 0.027, 95% CI [0.085, 0.852]) was found between AMT latency (time from TMS onset to AMT peak) and P200 within ROI1 (Figure 7). However, there were no significant correlations between MET, AMT latency, and P200 TEP in the ACLR group (Table 3).

### 3.3. TEP-Analysis

The mean amplitudes and latencies of a priori defined TEP components are presented in Table 4. In accordance with previous publications [43], TEPs could be identified in both groups. While the P30, N100, and P200 were identified in >80% of the subjects throughout all ROIs, the N45 and P60 components showed reduced subject inclusion rates.

**Figure 8:**
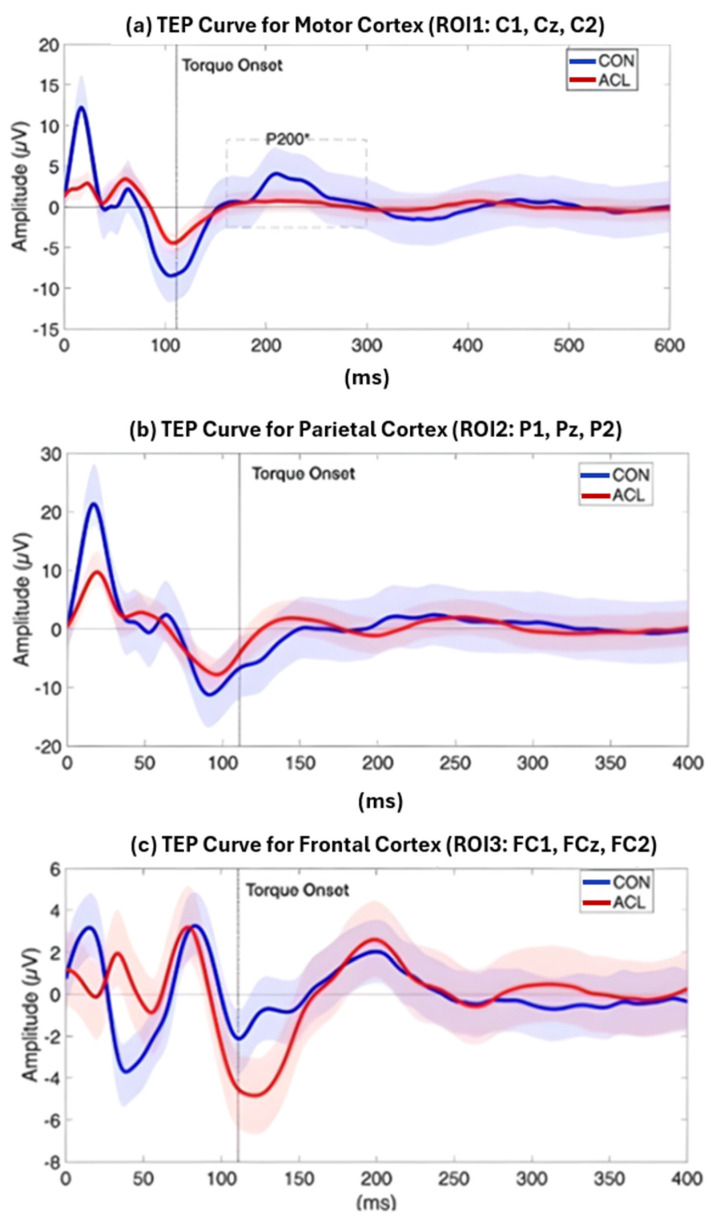
Grand average TEP curve for the ROI1 (**a**), ROI2 (**b**), and ROI3 (**c**) time locked to the onset of a TMS pulse at 120 and 140% MVIC, respectively. The red line displays the grand average TEP curve of sixteen ACLR, the blue line represents the grand average TEP curve of sixteen matched CONT, and the dashed vertical line displays the onset of TMS-evoked torque. Blue and red shades represent standard deviations of the TEP curves of the two compared samples, respectively. A significant difference (*) between ACLR and CONT has been observed for the amplitude of the P200 component (dashed box), which was attenuated for the ACLR group (*p* < 0.05).

**Figure 9:**
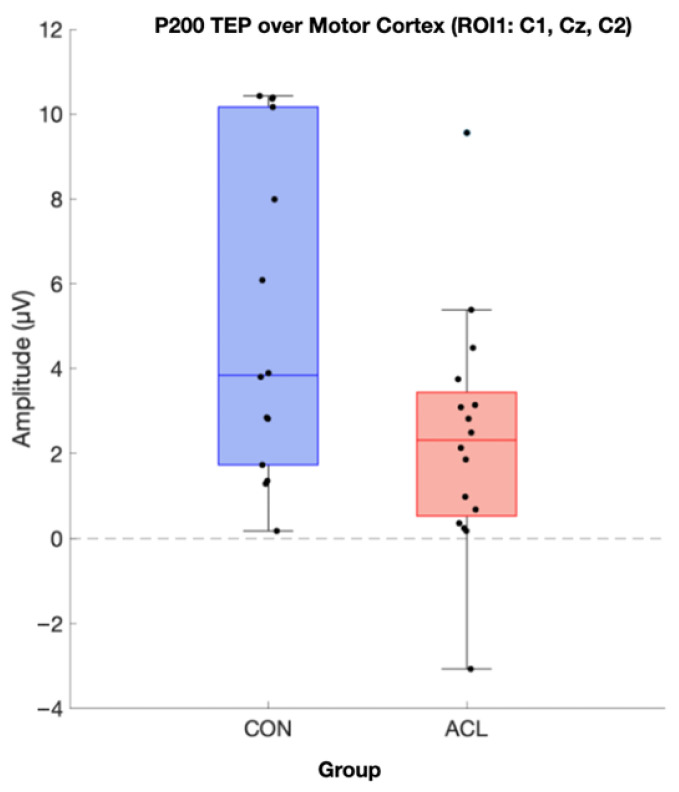
Boxplot comparison of TMS-evoked P200 amplitude of the two compared groups in the motor cortex (ROI 1). Sixteen CONT (blue box) and sixteen matched ACLR (red box) contributed to analysis. Data was extracted based on the statistics of the TESA toolbox. A significant difference for P200 amplitude has been observed (*p* < 0.05) (µV).

Permutation-based *t*-tests revealed significant group differences for the P200 component amplitude in ROI1 (*p* = 0.044, Cohen’s d = 0.854, 95% CI [0.25, 1.72]). Here, the ACL group demonstrated an attenuated mean P200 amplitude compared to the CONT group. A visualization of the grand average TEP in ROI1 highlighting the significant group differences can be found in Figure 8. Boxplots for the visualization of amplitude distributions can be found in Figure 9. TEP grand averages and associated topographical maps are displayed in Figure 4.

## 4. Discussion

The primary aim of this study was to explore potential neuroplastic changes in cortical excitability and motor cortex activation following anterior cruciate ligament reconstruction (ACLR). Our results displayed an increase in MET in the ACLR group following stimulus. Previous research has proposed that AMI contributes to decreased corticospinal excitability following ACLR, as altered afferent input from the injured ligament can lead to ongoing reflexive neural inhibition within the corticospinal tract to the muscles surrounding the knee joint. However, the elevated MET percentage change observed in our ACLR group suggests a heightened cortical response to TMS, potentially reflecting increased quadriceps contraction relative to healthy controls [6]. This finding supports the possibility of a corticospinal tract hyperactivation as a compensatory mechanism to overcome persistent AMI. Similar compensatory cortical hyperactivation to counteract residual quadriceps inhibition has been described in ACLR patients, whereas increased motor cortex activity is recruited to stabilize the knee and maintain quadriceps output [51]. While greater MET may enhance force output to reach similar pre-injury threshold muscle response [11], it could also reinforce altered motor control strategies with implications for long-term joint health and function. Excessive quadriceps activation without adequate hamstring co-activation may generate excessive anterior tibial translation, increase strain on ACL graft, and may influence ACL loading [52,53].

This study focuses on the mixed-cortical sensory components like P200 under experimental conditions, rather than exclusively on M1 excitability. Our results show that in participants with ACLR, there are differences in corticospinal tract and primary motor cortex activation during isometric force reproduction, with increased MET and reduced P200 amplitude in the central region of interest relative to CONT [23]. Alterations in behavioral outputs like MET indicate the development of neural changes that influence motor control in ACLR post-surgery. However, TMS-EEG literature suggests that early M1-TEP peaks saturate approximately in the first 50–60 ms post-TMS stimulation, comprising P30, N45, P60 responses, which most accurately reflect local motor and primary somatosensory inputs, with later responses, such as N100 and P180/P200, being strongly influenced by the somatosensory reafferent effects of the TMS pulse stimulation [23,27,28]. In our study, P30, N45, and P60 were consistently clearly distinguishable but did not show significant group differences in any of the regions of interest, with likely group differences existing in later sensorimotor processing [23,28].

The combined use of EEG and TMS in this study provided insight into electrocortical dynamics underlying these neuromuscular adaptations following ACL reconstruction. We observed a significantly lower P200 TEP, a central ROI covering the motor cortex, following TMS onset in the ACLR group. In TMS-EEG research, the P200, also referred to as P180, reflects a combination of late excitatory cortical reactivity, multisensory reafferent processing, and the propagation of TMS-induced activity through cortico-cortical pathways, rather than purely attentional or evaluative cognitive processes [30,31,43]. Reduced P200 TEP may therefore suggest attenuated neural processing within the primary motor cortex during force reproduction [54], which in turn could necessitate greater corticospinal drive, as reflected by the elevated MET in ACLR participants. It may suggest that reduced cortical excitability in ACLR patients, especially when evaluated in combination with the MET findings, requires recruitment of additional brain regions to compensate for motor cortex dysfunction during quadriceps activation [11]. For example, Chaput et al. (2025) [55] reported increased frontal activation during lower-limb tasks in individuals following ACL reconstruction, which may reflect enhanced involvement of distributed cortical networks involved in sensorimotor control strategies. This interpretation aligns with the understanding that late TEPs are not classical cognitive ERPs but rather index the integration of sensory and higher order cortical processing associated with adaptive motor control [23,28].

The presence of late TEPs in the 150–250 ms time range is thought to be affected by integration of sensory feedback and attentional requirements, such that differences in these components can assess modifications in sensorimotor control processing rather than purely motor or cognitive processing [23,28]. In support of this idea, neuroimaging and EEG studies have shown that subjects with an ACL reconstruction rely more on additional frontal and sensorimotor processing when engaging in lower limb tasks, indicating a change in processing toward attentional and feedback-dominant control [13]. Based on this rationale, a likely pattern in both P200 amplitudes and MET in subjects with an ACLR would seem to involve a processing modulation, which involves a compromised late sensorimotor processing stage with suppression over the activated motor area coupled with enhanced corticospinal activation in order to produce sufficient torque output during a sudden perturbation [13,23,28].

Given that an ACL injury can alter afferent sensory processing among different regions of the brain [13], the present study supports that ACLR patients possibly engage alternative neural mechanisms to exhibit proper motor function. An altered neuromuscular control strategy may prioritize sensory–motor feedback integration and cognitive spatial awareness rather than solely sensory feedback after ACLR [56,57]. Therefore, ACLR patients may transition from autonomic sensorimotor regulation toward more attention-dependent control strategies.

Grooms et al. (2017) [13] reported that ACLR participants exhibited greater motor cortex activation during simple knee flexion and extension tasks. Similarly, Baumeister et al. (2008) [15] observed greater frontal Theta-power, an indicator of focused attention during complex tasks, in ACLR. This may suggest a compensatory mechanism in which ACLR participants allocate greater cognitive effort to maintain task performance [58,59]. In our study, ACLR participants were instructed to maintain submaximal isometric quadriceps activation in response to unanticipated TMS perturbation, limiting reliance on visual cues and cognitive awareness. Thus, the reduced P200 TEP in our study suggest that the primary motor cortex in ACLR patients may not have been fully engaged, since regulation of the quadriceps after surgery often necessitates sensory, motor, and visual information together [22,54,55].

Notably, our study revealed that elevated MET was accompanied by reduced P200 TEP, a combination that may represent a maladaptive dissociation: compensatory corticospinal hyperactivation with attenuated cortical processing. This aligns with prior work reporting increased corticospinal drive following ACLR [13,60]. Such adaptations may initially support motor performance, but over time, they could reinforce inefficient neural strategies that contribute to quadriceps sensitivity, abnormal joint loading, and risk of re-injury. The correlation analysis between P200 TEP and active motor threshold (AMT) latency provided additional exploratory mechanistic insight. In the CONT group, P200 amplitude was moderately correlated with AMT latency, suggesting efficient coupling between cortical processing and corticospinal output. By contrast, this relationship was absent in the ACLR group. ACLR participants may exhibit disrupted feedback loops between sensory input and motor output pathways, potentially due to inhibitory mechanisms or altered afferent signaling [61,62]. Alternatively, the reduced variability in MET values within the ACLR group may have limited our ability to detect a correlation. Regardless, the absence of normal coupling highlights altered cortical–spinal integration following ACLR.

Collectively, these results could provide important clinical implications. The observation of heightened corticospinal excitability (MET) and reduced cortical responsiveness (P200) may suggest that ACLR patients may rely on compensatory neuroplastic mechanisms that could support short term motor function and contribute to abnormal neuromuscular control. Rehabilitation strategies should therefore aim not only to restore strength but also to normalize sensorimotor integration and cortical activation patterns. Interventions such as neuromuscular electrical nerve stimulation and cryotherapy can be utilized to help mitigate AMI [6], while electromyographic (EMG) biofeedback could support motor cortex processing by allowing patients to regulate quadriceps activation in real-time [63]. Moreover, integrated cognitive–motor rehabilitation—combining proprioceptive and cognitive training and task-specific strengthening—may reduce reliance on compensatory cortical hyperactivation and promote more efficient neural control strategies [2,63]. Finally, the combined use of EEG and TMS provides a valuable neurophysiological framework for evaluating the efficacy of these interventions and monitoring neural recovery trajectories post-ACLR.

### Limitations

Side-to-side quadricep strength deficits greater than 10% have been linked to decreased self-reported function, reduced physical performance, and an elevated risk of reinjury due to altered knee biomechanics [64]. In the present study, however, quadriceps strength was only assessed in the reconstructed knee for the ACLR group and the matched limb of the CONT group. This unilateral assessment limits interpretation, as bilateral measurements of MET and TEP would provide more comprehensive information about neuroadaptations at both the motor cortex and corticospinal tract following ACLR. Such insights could help guide the development of more targeted rehabilitation techniques aimed at reducing re-injury risk [55]. Similarly, bilateral measurements in QMVIC may help determine the extent to which the non-injured limb contributes to compensatory motor strategies, thereby revealing neural differences between injured and non-injured legs [55]. These compensatory behaviors are relevant to consider, as they may underlie biomechanical issues such as gait alterations, which can influence the optimization of rehabilitation therapy [65].

Another limitation relates to the intensity and limited number of TMS pulses at 120% and 140% AMT in both ACLR and CONT groups. There were only 30 trials taken per intensity for this exploratory study because of participant fatigue. To maximize participant engagement while ensuring reliability, a minimum of 80 trials should be considered for future studies [25]. TMS intensity of 120% and 140% may have produced effects beyond the targeted region of the motor cortex in this study. Future studies may consider a lower TMS intensity of 110% to ensure targeted stimulation [25].

To achieve an adequate sample size for TEP analysis, TMS pulses across AMT intensities were pooled for ACLR and CONT, and the mean of all 60 trials was analyzed. Thus, TMS stimulation intensity could not be used as a variable due to the limited number of trials per condition. Because motor cortex excitability and recruitment of neuronal populations vary with TMS intensities, combining data across intensities may mask nuanced differences in cortical excitability [26,66,67]. Furthermore, utilizing a two-way ANOVA to examine the effects of stimulation intensity, at 120% and 140% AMT, on both ACLR and CONT groups would likely have provided a more comprehensive analysis than the independent samples *t*-tests used in the current study.

TEPs, especially the N100 and P200 components, can be particularly affected by auditory and somatosensory ERPs that are evoked by the clicking of the TMS coil and stimulation of the scalp. However, recent studies have demonstrated that a full protocol of sensory suppression can minimize these late components effectively with minimal effects on early M1 peak amplitudes [28]. In this study, earplugs and independent component analysis (ICA) were used to reduce sensorimotor artifacts, but active noise cancelling or foam padding were not used, such that N100 and P200 components are considered composite sensorimotor responses, which can no longer be considered direct indices of local cortical excitability [23,28,68]. While earplugs may reduce the volume of the TMS click, they do not mask auditory processing as effectively as white noise delivered through headphones. Although Beck et al. (2024) [27] highlights that noise masking does not completely eliminate auditory co-stimulation, it is an important control measure to improve the validity of TMS-EEG studies and should be included in future studies.

Given our use of earplugs but no full noise-masking, P200 in this study should be interpreted as a composite sensorimotor response rather than a “pure” M1 indication. Furthermore, despite identifying P30, N45, and P60 components in both groups, these did not show a significant difference in both ACLR and control subjects, which suggests that our primary results are not sufficient in deriving conclusive results concerning early M1 TMS activation but rather late composite sensorimotor processing in both patient and control subjects [27,28,69]. Additionally, recent experimentation found that sensory suppression procedures can markedly reduce N100 and P200 components while also sparing early components, minimizing noise [68]. These suppression procedures should also be further explored and considered in future experimentation.

Additionally, our grand-average TEP visualization displayed in Figure 4 revealed the presence of residual artifacts. These observed patterns differ from the more spatially coherent and temporally standard M1-TEP reported in studies that employ aggressive artifact suppression pipelines, such as described by Lucarelli et al. [25]. Accordingly, the present TEP results likely reflect a mixture of neural and non-neural contributions, which may have reduced reliability to group differences. For a more externally valid analyses, future studies should incorporate more trials to effectively get rid of systematic TEP artifact.

Furthermore, ROIs in this study were not defined by component-specific scalp topography in each time window, as recommended in recent TMS-EEG [25,26]. Instead, we used fixed, midline, anatomy-based ROIs centered on M1 and adjacent cortices to test a priori hypotheses about late N100/P200 sensorimotor processing during a force reproduction task. While this conservative strategy reduces circularity, it may underestimate effects that peak outside the predefined clusters. Future studies with larger samples and more trials should complement this approach with data-driven, topography-based ROI selection and source analyses.

RTTpeak collected at 100% intensity as the normalization factor for MET may serve as another potential methodological limitation. M-wave Maximum (M-Max) is often used as a standard for normalization in similar studies [70]. The decision to use RTTpeak at 100% intensity for normalization rather than M-Max was made due to the difficulties of normalizing to M-wave maximum (M-max) with a small study population. It may be beneficial, however, for future studies to consider alternative normalization methods, such as normalizing to M-max, for more accurate comparisons.

The greater MET may have resulted from multiple factors besides corticospinal hyperexcitability, including changes at the spinal level. This study did not measure the Hoffmann reflex using EMG and electrical stimulation, which may provide neuromuscular information necessary to understand neuroplasticity at the corticospinal tract after an injury [71]. H-reflex measured during voluntary contraction of the muscle, or during the specified task, is most effective in eliciting a quantifiable change in the reflex amplitude [71]. While the MET data from this study suggests corticospinal hyperexcitability, measures such as EMG and H-reflex would be necessary to determine the precise activity of the muscle in contributing to cortical excitability [18].

Finally, it is not possible to determine whether the observed neural changes observed in the ACLR participants were a direct result of the injury as no baseline measures of corticospinal activity prior to ACL injury were conducted. Future longitudinal studies that incorporate early-post-injury assessments would help clarify the trajectory of these adaptations.

## 5. Conclusions

The primary contribution of this study was the behavioral MET findings, as the preliminary ERP data remains exploratory in nature. This study revealed altered activation patterns in the corticospinal tract pathway during submaximal quadriceps isometric contraction in individuals with ACL reconstruction (ACLR), characterized by elevated MET in response to unanticipated TMS pulses. The change in corticospinal tract excitability may contribute to persistent quadriceps impairments. While the relationship between motor cortex and corticospinal excitability remains unclear, our behavioral findings suggest that neuroadaptive responses in the corticospinal tract play a significant role in post-surgery motor control. Although preliminary, the brain data may be used for future hypothesis-driven research aimed at understanding the relationships between the corticospinal tract, motor cortex, and sensory areas of the central nervous system after injury.

The results highlight the importance of studying neuroplastic changes after injury, as it may help inform effective post-injury care options. Traditional ACL rehabilitation programs primarily focus on strengthening and restoring the full range of motion as the first and most important protocols following surgery. However, our study suggests that earlier interventions targeting neuromuscular recovery may offer additional benefits. Research indicates that neurocognitive and cognitive motor training, such as dual task movement, visuomotor feedback, or task-oriented motor learning, can improve corticomotor efficiency, enhance proprioceptive integration, and restore neuromuscular patterns [71]. Patients would likely benefit from earlier neuromuscular healing strategies, as sensory information is affected immediately after surgery. H-reflex measures, for example, can provide information on neuromuscular plasticity in ACLR, which can guide these rehabilitative efforts. Although strength and mobility restoration are essential parts of post-injury care, ACL rehabilitation programs should also utilize neurologic and neurocognitive strategies that optimize motor function and reduce the risk of re-injury. A goal-directed processing intervention may be beneficial in improving neuromuscular control for ACLR patients. The elevated MET, particularly when interpreted alongside the reduced P200 peak power, suggests that a combination approach to rehabilitation that is targeted at both cognitive and motor processes may be beneficial. Cognitive rehabilitation, focused on setting goals and redefining existing thought patterns, along with structured motor control activities like balance training, may resolve the motor and perceptual deficits that patients experience after surgery. Implementing cognitive and motor skills through goal-directed therapy can restructure how ACLR patients think about and approach their recovery [1,2].

The behavioral findings in this study can be used as a foundation for additional research on neuroplastic changes in the corticospinal tract and motor cortex. Combining TMS-EEG with MET and EMG findings in hypothesis-driven research could offer a more comprehensive understanding of quadriceps function and neuroplasticity, and inform more targeted, mechanism-based interventions. Future studies should include bilateral assessments to better understand strength deficits and compensatory behaviors that ACLR patients develop over time. Additionally, expanding the sample size would permit the use of more robust statistical analyses, enabling a deeper understanding of groups and condition-specific patterns. Longitudinal studies may provide a baseline assessment to gauge whether neuroadaptation occurred after injury or after surgery in ACLR participants. Lastly, performing longitudinal studies in older populations may offer insight into the long-term effects of reconstructive surgery and the relationship of MET, brain activation patterns, and rehabilitation with aging.

## Figures and Tables

**Table 1:** Characteristics of Study Population. Note: ^+^ One participant had two reconstructive surgeries on the same knee (quadricep and patellar tendon). Age, weight, and height are written as mean ± standard deviation. ^☨^ Mean rank from Mann–Whitney U test for nonparametric comparisons. * Denotes significant group differences (*p* < 0.05).

Variables	Control (*n* = 16, 10F, 6M)	ACLR (*n* = 16, 10F, 6M)	*p*-Value
Age (years)	20.6 ± 1.4	20.0 ± 1.2	0.296
Weight (kg)	66.3 ± 11.0	75.8 ± 17.1	0.211
Height (cm)	168.0 ± 9.9	171.9 ± 7.2	0.078
Time Since Reconstruction (months)			
4–12	-	6	-
13–20	-	1	-
21–30	-	2	-
>30	-	7	-
Graft Type			
Patella Tendon	-	5	-
Hamstring	-	6	-
Quadriceps	-	2	-
Autograft	-	2	-
^+^ More than One Type	-	1	-
Knee Function Outcomes			
IKDC ^☨^	24.16	8.84	<0.001 *
Lysholm ^☨^	23.50	9.50	<0.001 *
Tegner ^☨^	17.53	15.47	0.525

**Table 2:** Quadriceps Strength Outcomes (Mean ± SD). Table Abbreviations: QMVIC: Quadriceps Maximum Voluntary Isometric Contraction; QMVIC_norm_: QMVIC normalized to body weight; QMVIC_10%_: 10% of QMVIC; AMT_%MSO_: minimum TMS intensity to produce largest and consistent METs; MET_10%QMVIC_: MET peak torque amplitude to TMS stimuli; MET_peak_: MET absolute MET torque increase from QMVIC_10%_; MET_lat_: MET peak torque latency; RTT_peak_: peak resting twitch torque; MET_norm_: Motor evoked torque normalized to RTT_peak_.

	CONT	ACLR	*p*-Value	Effect Size (d)
QMVIC (Nm)	180.44 ± 58.78	198.43 ± 63.94	0.414	0.29
QMVIC_norm_ (Nm/kg)	2.72 ± 0.73	2.66 ± 0.78	0.831	−0.08
QMVIC_10%_ (Nm)	17.44 ± 6.35	19.88 ± 6.42	0.289	0.38
AMT_%MSO_ (%)	44.69 ± 10.18	39.77 ± 5.20	0.104	−0.60
MET_10%QMVIC_ (Nm)	26.14 ± 7.23	32.12 ± 6.84	0.023 *	0.85
MET_peak_ (Nm)	8.08 ± 3.52	12.24 ± 3.76	0.003 *	1.14
MET_lat_ (ms)	115.04 ± 9.38	113.25 ± 11.05	0.626	0.52
RTT_peak_ (Nm)	9.69 ± 5.15	7.62 ± 3.51	0.194	0.47
MET_norm_ (%)	90.08 ± 39.63	188.97 ± 85.94	<0.001 **	1.48

A * denotes a significant difference between groups (*p* < 0.05), ** denotes a significant difference between groups (*p* < 0.001).

**Table 3:** Spearman’s correlations between MET_norm_, MET_lat_, and motor cortex activation (ROI1 TEP). * Denotes a significant correlation (*p* < 0.05).

	CONT			ACLR		
	MET_norm_	MET_lat_	P200	MET_norm_	MET_lat_	P200
MET_norm_	-	0.125	−0.053	-	−0.063	−0.151
MET_lat_	-	-	0.587 *	-	-	−0.089
P200	-	-	-	-	-	

**Table 4:** Mean amplitudes and latencies of a priori defined TEP components.

		CONT Amplitude (Mean ± SD)	CONT Latency (Mean ± SD)	ACLR Amplitude (Mean ± SD)	ACLR Latency (Mean ± SD)	CONT % (*n*)	ACLR % (*n*)	Pperm Amp	Pperm Lat
ROI1 (C1-Cz-C2)	N100 P200	−11.26 ± 11.28 6.44 ± 4.93	120.25 ± 34.08 225.13 ± 31.84	−6.01 ± 5.74 2.38 ± 2.79	127.63 ± 31.19 212.75 ± 36.29	87.50 (14) 87.50 (14)	100.00 (16) 100.00 (16)	0.460 0.044 *	0.546 0.316
ROI 2 (P1-Pz-P2)	N100 P200	−16.96± 21.29 6.27 ± 5.57	106.75 ± 29.26 239.25 ± 41.13	−13.06 ± 12.55 4.64 ± 3.56	120.38 ± 41.05 236.63 ± 42.49	87.50 (14) 87.50 (14)	87.50 (14) 100.00 (16)	0.741 0.961	0.934 0.871
ROI3 (FC1-FCz-FC2)	N100 P200	−6.08 ± 4.77 4.09 ± 2.72	126.25 ± 23.48 210.25 ± 40.00	−6.90 ± 4.53 3.89 ± 3.32	114.25 ± 21.99 215.63 ± 34.70	100.00 (16) 100.00 (16)	100.00 (16) 100.00 (16)	0.626 0.852	0.608 0.955

Pperm Amp: Permutation-based *t*-test for mean amplitude, Pperm Lat: Permutation-based *t*-test for mean latency. Values in columns 7 and 8 of the table reflect the percentage of participants included in the analysis. A * denotes statistical significance between groups (*p* < 0.05).

## Data Availability

The data presented in this study are available on request from the corresponding author due to privacy restrictions.

## Abbreviations

The following abbreviations are used in this manuscript:

ACL	Anterior Cruciate Ligament
ACLR	Anterior Cruciate Ligament Reconstruction
CONT	Control
TMS	Transcranial Magnetic Stimulation
EEG	Electroencephalography
QMVIC	Quadriceps Maximal Voluntary Isometric Contraction
MET	Motor-Evoked Torque
MET_norm_	Motor-Evoked Torque Normalized
ROI	Region of Interest
TEP	TMS-Evoked Potential
CI	Confidence Interval
MEP	Motor-Evoked Potentials
AMT	Active Motor Threshold
ERP	Event-Related Potential
IKDC	International Knee Documentation Committee
RTT	Resting Twitch Torque
FastICA	Fast Independent Component Analysis
EMG	Electromyographic
AMI	Arthrogenic Muscle Inhibition
M-Max	M-wave Maximum
